# Stakeholder involvement in health research priority setting in low income countries: the case of Zambia

**DOI:** 10.1186/s40900-018-0121-3

**Published:** 2018-11-05

**Authors:** Lydia Kapiriri

**Affiliations:** Department of Health and Aging, McMaster University, 1280 Main Street West, Hamilton, ON Canada

**Keywords:** Health research priority setting, Low income countries, Stakeholder involvement, Zambia

## Abstract

**Summary:**

While there is increasing recognition of the importance of stakeholder involvement in health research priority setting there is a paucity of literature reporting on stakeholder involvement in health research priority setting in low income countries. This paper fills this gap by identifying and discussing the roles and legitimacy of different stakeholders (including the public and patients) involved in the health research priority setting process in Zambia; identifying the barriers to public participation and proposing improvement strategies.

We interviewed 28 policy makers and practitioners who had participated in the national level health research priority setting in Zambia. Reported participants in health research priority setting included research users, researchers, research funders and the community/ public. Research funders were thought to have undue influence while the public and patients were not effectively involved. This could be due to the public’s lack of education, lack of resources to facilitate public involvement and limited skills to meaningfully engage the public. Participation of people from rural areas, women and young professionals was also limited.

While there is a commitment to broad stakeholder involvement in health research priority setting, there’s limited public/patient involvement. Public education, availing more resources, and skills to meaningfully engage the public need to be explored. The undue influence of research funders should be mitigated and incentives availed to ensure that they align their research funding with the national priorities. These efforts would strengthen meaningful stakeholder engagement in health research prioritization within Zambia and other similar contexts.

**Abstract:**

**Background** Stakeholder involvement in health research priority setting contributes to the legitimacy and acceptability of the priorities. Hence legitimate priority setting should involve a broad representation of stakeholders including the public. While there is a growing body of literature on health research prioritization in low income countries, there is a paucity of literature reporting on stakeholder involvement in the process. The objectives of this paper are to; 1) identify the stakeholders who were involved in the health research priority setting process in Zambia; 2) discuss the roles and perceived legitimacy of the stakeholders and analyze the degree to which patients/ public was involved; 3) To discuss some of the barriers to stakeholder participation in Zambia and similar contexts and to propose improvement strategies.

**Methods** This was a qualitative study involving 28 in-depth interviews with stakeholders who had participated in the national level health research priority setting exercises in Zambia. An interview guide was used. Audio recorded interviews were transcribed and analyzed using INVIVO 10. Analysis of the Stakeholders’ theme involved identifying the different dimensions of stakeholder involvement as discussed in the interviews.

**Results** Identified stakeholders included; research users, researchers, research funders and the community/ public. We found that health research priority setting involved research users, researchers, research funders and the community/ public. However, research funders were thought to have undue influence while the public and patients were not effectively involved. While the respondents recognized the advantages of involving the public and patients, they were not effectively involved. This could be due to the public’s limited understanding of the technicalities of priority setting, lack of resources to facilitate public involvement and limited skills to meaningfully engage the public. Participation from rural areas, women, and young professionals was also limited.

**Conclusions** While there is a commitment to broad stakeholder involvement in health research priority setting, the public is left out. Efforts such as public education, availing more resources, and skills to meaningfully engage the public need to be explored. The undue influence of research funders should be mitigated through their direct involvement in the prioritization process and incentives to ensure that they align their research funding with the national priorities. These efforts would strengthen meaningful stakeholder engagement in health research prioritization within Zambia and other similar contexts.

## Background

Priority setting for health research is an important component in evidence based health policy making. This is because there are finite health research funds and numerous health research themes and questions that could potentially be funded to facilitate evidence based decision making within the health sector [1, 2]. Since these choices may result in winners and losers, it is important that priority setting is participatory, involving a broad representation of the relevant stakeholders.

Involving relevant stakeholders in priority setting can increase the legitimacy, credibility, acceptability and ownership of the decisions that are made [3]. Furthermore, involving stakeholders in priority setting for health research can contribute increasing the contextual relevance of the identified priorities. It also improves chances for uptake and use of the generated evidence. Bringing the relevant stakeholders at the same table may also reduce duplicity and the corresponding resource wastage [4].

The relevance of stakeholder engagement in priority setting is highlighted in most of the approaches/ frameworks that guide health research prioritization processes, such as; The Essential National Health Research Strategy (ENHRS) Framework [5]; The Combined Approach Matrix (CAM) [6]; Child Health and Nutrition Research Initiative (CHNRI) Framework [7]; The James Lind Alliance (JLA) PS Framework [8]; Listening for Direction (L4D) Framework [9]. Table 1 summarizes the details of the stakeholders identified within the different approaches.

**Table 1:** What do the common approaches to health research prioritization say about stakeholder engagement?

*Approach*	*Specifics about the recommended stakeholders*
The Essential National Health Research Strategy (ENHRS)	Categories of stakeholders identified include; researchers, decision makers, health service providers and communities.
The Combined Approach Matrix (CAM)	Participants include: individuals, households and community; health ministry and other health institutions; other sectors apart from health; and macroeconomic level actors.
Child Health and Nutrition Research Initiative (CHNRI) Framework	Specifically identifies various key stakeholders: PS framework managers or initiators, a group of experts, called in some cases a Technical Working Group (TWG) and a Larger Reference Group (LRG)—featuring wide stakeholder representation—to assess the importance of the criteria.
The James Lind Alliance (JLA) PS Framework	Identifies a priority setting partnership/ steering group which includes: Patients affected by the disease/condition/treatment need; care givers assisting those suffering from the disease or condition; and clinicians.
Listening for Direction (L4D) Framework	Describes several stakeholders: A group of experts who determine the “desired mix of key stakeholders” who should participate in the initial consultation--- mostly decision-makers.

Ref: [5–9]

While these approaches have been used to guide health research prioritization, there has been limited systematic analysis of stakeholder engagement in health research priority setting using these approaches or in other exercises. Yoshida et al., 2016 assessed stakeholder engagement in cases where the CHNRI approach was used. They found that there was a wide variation in the number of people included in the wide reference group, and what the stakeholders were required to contribute. They also highlighted the importance of involving people who have experienced the health issue (s) that are being considered [10]. While Tomlinson et al., 2011 summarizing different country experiences reported a lack of genuine stakeholder engagement [11].

Some of the other literature has identified the stakeholders that should be involved such as: researchers, funders, policy makers, government, civil society organizations, patients and the public. This literature also emphasizes the need for diverse representation of: disciplines, expertise, gender and sectors (beyond the health sector) [4, 12]; industry and ethicists [11]. However, an emerging theme in the literature on stakeholder engagement are the challenges associated with stakeholder engagement, generally and community participation, specifically [2, 10, 11, 13]. These issues have not been empirically explored within the context of low income countries.

Zambia, a low income country, is committed to participatory decision making. The country has conducted several health research priority setting exercises [14], however, there has not been any systematic evaluation of the degree to which these processes have been participatory. While there are reports and literature describing and evaluating their priority setting process [14, 15], there lacks an in depth analysis and understanding of the degree to which the relevant stakeholders, especially the public, were engaged in the health research prioritization process in Zambia. Questions such as: To what degree was the approach and process able to achieve broad stakeholder engagement? Who was involved in the process? What role did they play? And most importantly how did the patients and public participate (if at all)? Are yet to be answered.

This paper, responds to the above questions; recognizing the importance of stakeholder and the paucity of literature that addresses this critical issue. While it is based on experiences from a low income country the findings are, arguably, of relevance to any researchers, policy makers and stakeholders interested in participatory health research prioritization.

Study objectives are to;

1. Identify the stakeholders who were involved in the health research priority setting process in Zambia.
2. Assess how and the degree to which patients/ public have been involved in health research prioritization.
3. Identify the barriers to patients/public participation and discuss strategies through which they can be overcome.

## Methods

### Setting

The study was conducted in Zambia. Zambia has a decentralized health system, where the national level is responsible for policy making and the sub- national levels (provinces and districts) are responsible for policy implementation and service delivery. Respondents were drawn from the national and provincial levels.

### Sampling

We employed purposive and snowball sampling strategies. Respondents were recruited by virtue of their having participated in at least one health research priority setting exercise in Zambia. Since the general public did not participate in this process, they were not sampled. First, we obtained a list of people who had participated in at least one health research prioritization process in Zambia. These were initially contacted via e-mail. Consent to participate was registered when they completed and returned the written consent. Once they had been interviewed, they were requested to forward the invitation e-mail to any additional potential respondents. We stopped identifying respondents once the forwarded e-mails ended up consistently with respondents we had already interviewed.

### Data collection

Key informant interviews were conducted between 2015 and 2016. An interview guide, based on an evaluation framework with parameters for successful priority setting, was used. One of those parameters was stakeholder engagement in the priority setting process (Kapiriri, Forthcoming). This question explored issues such as who is involved, how and why are they involved and specifically what role (if any) is played by the public? Face to face interviews were conducted by a trained research assistant. The interviews lasted an average of 40 min and were audio recorded by permission from the respondents. The recorded interviews were transcribed verbatim.

### Analysis

The analysis was conducted by two researchers. They independently read through two transcripts and identified broad themes. One of the themes was stakeholder engagement. Further analysis for this paper involved: *(i)* micro-coding within the stakeholder engagement theme, whereby each researcher read through the stakeholder theme and identified codes which were given names. After which, the researchers met and compared their initial coding and code names. They discussed and agreed on code names which were used as a basis for coding the rest of the interviews using NVIVO 10*. (ii)* At an abstract level, the related code names were grouped together into categories. *(iii)* Related categories were grouped together to form broad themes which described different dimensions of stakeholder engagement such as; stakeholder names, stakeholder relevance, stakeholder role, stakeholder mode of engagement, barriers to participation. The results section is organized according to these dimensions of stakeholder engagement that emerged from the interviews. Where appropriate, illustrative quotes are used with the respondents identified with [numbers].

## Results

A total of 28 interviews were included in this analysis. Most of the respondents were either policy makers- working with the health ministry (7); or researchers- working at the university (7) or with research funding agencies (6). The rest of the respondents came from either the province, research institutes, other government ministries and non- government organizations.

All respondents agreed that health research priority setting *should be* participatory and involve a wide range of stakeholders; with some reporting that this has been key in their priority setting exercises.

*“At the priority setting exercise all the stakeholders are invited. So, it wouldn’t be just researchers, it would be the policy makers, people in the community. So, for example if you’re looking at priority setting for research on children, you will have children at these workshops or meetings. And all participants are encouraged to contribute” [#24*].

### Who is involved in health research priority setting in Zambia?

Respondents identified several stakeholders that participate in health research priority setting. These could be grouped under four broad categories; (i) research funders (including bilateral agencies/ donors, the world bank, ministry of Finance); (ii) Research users (policy makers (national, province and donors), practitioners- health providers, donors); (iii) researchers (at academic and research institutes); (iv) Research Beneficiaries (the community). We discuss these groups and their reported roles below.

### Research funders

Research funders included the government (the ministry of finance), and donors (including the World Bank, and several bilateral organizations). These were identified as key stakeholders, by virtue of their having the funds to facilitate research. While they were recognized as key stakeholders, there was concern that sometimes donors leave the identified research priorities and fund research topics that are not part of the priority list.

*“…Of course, you can’t leave out the funders. Research is very expensive. You may know the need, you may have the problem, you may have the know-how of how to address this issue, but you need the funding…” [#3].*

“…A*s implementers, we have the authority because we have the evidence and all that, and would want to have influence but we don’t have the funds. So, you would find that donors might influence and push agendas which might not even be priorities because they’ve got funds…”[#1].*

#### Research users

Respondents identified several categories of research users including; government (national and provincial policy makers), health practitioners, non-government organizations, and the community.

“*…Probably the policy makers, the policy makers… consume the product of that research. The service providers could be government, or could be the NGO sector, and government organizations also listen, understand, and then they implement.”* [#21].

##### Policy makers

The policy makers within the ministry of health were identified as key users of the generated research evidence when making policy decision making. Respondents identified the ministry of health as the legitimate facilitator of the health research prioritization process who should ensure that the process is participatory, transparent, credible, consistent, feasible and that the implementers (funders and researchers) do not deviate from the identified priorities.

*“So, I guess the Ministry of Health is the top tier, and is the coordinating body for health research in Zambia. So they are ones who basically initiate the whole priority setting process, bringing stakeholders on board and saying, Okay, we’ve got this disease area. What are the priorities? Can we establish them so that anybody who wants to do health research buys into this list of priorities that we have?” [#22].*

*“The roles that they (the policy makers) play is to guide us … to set the right priorities. So, I think that’s the main role they play… making sure that we don’t deviate and set priorities which are… not priorities at the national level and also that we set priorities that are realistic, practical, and can be achieved.” [#24].*

*“So, their (government policy makers) job is to make sure that the right priorities are set, the process of coming up with the priorities are transparent, they are accountable, and they build on broad consultation and, you know, communicate with all the key stakeholders.” [#26].*

While they identified policy makers as a general category, some of the respondents also highlighted the need to ensure that knowledgeable people take the lead and are involved in the process. Among the stakeholders who were identified as knowledgeable were health practitioners.

##### Health practitioners

The respondents discussed that health practitioners as key stakeholders in health research priority setting, based on their two-fold perceived roles; *(i)* users of the research findings, and *(ii)* key representatives of the interests of the patients and the public.

As users, many respondents felt that research priorities should be established to help frontline workers and to improve the health of individuals. This can only be achieved by hearing the voice of those that are on the “ground”, treating the patients:

*“What I’m driving at is that we should have workers or even practitioners… if we are to improve services based on research… practitioners who are conscious of certain things that are happening, right?” [#11].*

*“Cause essentially they are implementers. They are on the ground. They identify some of these issues and they come and they talk to us…” [#25].*

However, while some respondents thought that health practitioners participated in previous priority setting processes, a few respondents had contradictory perspectives with regards to the role that the practitioners *actually* played in health research prioritization. These thought that the limited role played by practitioners, local experts and Non-government organizations (NGOs) was a major weakness in the PS processes that they participated in.

### Researchers

Researchers were also identified as key players in the prioritization process. Respondents discussed that researchers as experts who had the time to produce the evidence, and present it to the policy makers and politicians in an understandable and “attractive” form.

*“… Researchers can look at the evidence so they know where the problems are and they have the knowledge to do something about it, so they have a role…”* [#27].

*“… In my view, most of the research should be done in academia. PS should mainly be driven by the academics, because they have time, in most cases, to do research…”* [#4].

*“…The researchers themselves, they have to produce research that is convincing enough for the politician to be attracted to make decisions...” [#11].*

While there was general agreement about the critical role played by the researchers, there were varied perspectives with regards to their actual involvement, and the undue influence they might have on the process. While some researcher respondents felt “used” to simply provide the information:

*“…So, our role in that process was really technical. To supply the information as requested and then the Ministry of Health takes it up into implementation…” [#26].*

However, non-researcher respondents thought that researchers actually had too much influence due to their research expertise:

*“…You would expect that the policy makers would have a big influence because they all set the tone as to what happens to those research results, but unfortunately that’s not the case. [I see.] Yes, because the researchers are the ones who, in most cases, drive the whole research evidence production including the publication and dissemination. So they’re in a position of advantage because they have the knowledge and the information to speak on what should be or what should not be (done)..*.” *[#19].*

Interestingly, researcher respondents discussed their frustrations with donor funding which may not always reflect the local priorities and capacities;

*“…Someone has money to fund your priorities number eight. Can you say, no we must start with number one? No. You let him go ahead with funding number eight, which is not necessarily your priority number one. And that is what has been happening. And so for the researchers it has been very frustrating, you know, to put up proposals after proposal, and nobody funds your proposals. And so most of the time you just have to sit and wait. But as I said, you know, the funding that comes from abroad has its own research agenda. And so our local researchers end up being more like research assistants because they are not there where the agenda is being set. And that, I don’t think, is the best way of doing things. If we have to have research priorities, there must be funding to carry them out, to implement those priorities…” [#9].*

### Research beneficiaries

Research beneficiaries (including members from the community, (public and/or patient groups)) were recognized as key to health research prioritization, as was illustrated by [#24]. Respondents felt that people who are most affected by the priority setting decisions ***should*** participate in the process.

“…*I have a strong belief that because I’m a public servant and I believe in whatever we’re doing, if we’re going to call it a national thing, the main stakeholders should always be the people who are you are going to save or the people in which that research is going to be conducted. So the civil society (organizations) or the communities, the people, the public are the number one main stakeholder…” [#9].*

Respondents discussed several reasons why the public should be involved in health research priority setting. Public participation was thought to increase the chances for; identifying relevant topics, success, adding an ethical perspective, and responding to community needs. Communities were thought to be useful in identifying health issues that specifically affect their communities; based on which priority research questions can be formulated. These are summarised in Table 2 with demonstrative quotes.

**Table 2:** Benefits of public engagement in health research priority setting with illustrative quotes

Reason	Quote
Increased chances of identifying research topics relevant to society	“…I believe that research should be relevant to the needs of society. Yeah. So those are the main ones, because you do research for them.” [#7]
Increased chances of success	“Ensuring that you have incorporated the views of the various important stakeholders. And in this case the key stakeholders are usually the… are usually the people on the ground - the communities, the civil society. And… and if you can engage them in identifying what priorities you should be looking at, then you are successful.” [#9]
To gain an ethical perspective	“The community leaders will basically bring in the ethical perspective.” [#3]
To prevent future disagreements	“…So public should feel they participate because when you set priorities as government and they come out as a priority, the public starts arguing that no these are not. It distorts your planning. There’s always this tug of war with the people you feel … So they should be involved that’s what I’m saying…”[#2]
Identify topics that respond to community experiences	“I think communities, participants need to have a say in research priority setting because they are the ones that experience whatever problems they have so they need to have a say.” [#27] “So, many of their priorities would actually come from the various experiences they have had, rather than them having read certain things and so on.”[#3]

### Community engagement strategies

Respondents identified several mechanisms through which the community has been involved in health research priority setting. These included; conducting community based research/ needs assessments, consultations, and through representatives. A few respondents cited one occasion when the public was consulted in identifying their specific research priorities;

*“… I mean the previous priority setting when we completed the list and launched it, the same week a group of people came complaining that their priorities were not included. And these were the marginalized groups. So, the last [priority setting exercise], we went specifically for the marginalized communities and we did some research to identify what are their health priorities” [#1].*

“…*we just have to go down to the grass root, and involve them in the setting of these priorities…” [#23].*

However, while a few respondents thought this group was involved through direct consultations; the majority thought they were involved through representation---mainly by local health practitioners (discussed above) civil society organizations (CSOs), and politicians.

With regards to CSOs, while the respondents recognized their critical role since they are perceived to be in direct contact with the community;

*“…we involve those people (CSOs) because they are on the ground, they interact with the community, and they know what the needs are in that particular area...” [#13].*

The respondents also discussed some of the limitations of working with the CSOs since they tended to represent different community groupings within society, which increases the resource needs for their mobilization, sensitization and education [#29]; in order to facilitate broad representation.

Politicians were also identified as legitimate representatives of the communities, since they are elected representatives from the communities they represent who have the privilege of interacting with policy makers;

*“I would feel that maybe the politicians, especially the Members of Parliament* ***should be*** *involved in priority setting because they interact with the (grassroots) as well as the (government officials). They are our leaders in every constituency, they live among the people …” [#3].*

While politicians were identified as legitimate community representatives, this seemed aspirational, since most of the respondents did not identify them as having participated in the priority setting exercises; instead, technocrats from other government ministries were represented in the priority setting process.

When asked about the degree to which the public/ community was ***actually*** involved in health research priority setting; the responses varied. There was a perception, by a few respondents, that the community was involved in stages of the prioritization process; *“…They were involved in all stages. They’re involved in contributing their experiences and what they think are some of the priorities that should be looked at…” [#9].* However, most of the respondents discussed that the community/ public was missing in the health research prioritization process;

*“…But I think the missing link, are the beneficiaries of research – basically the communities - there was no community representation. And I think that definitely is a gap…” [#19].*

### Barriers to public participation

First, some of the respondents reported that there was a perception- among the technocrats-- that the public may not be able to make meaningful contributions to the prioritization process, which is perceived to be technical. These doubted the public’s ability to understand priority setting and its technicalities.

*“… you know, we always take it for granted that the community don’t have probably much to contribute at the technical level but I think that was a gap because we needed the community perspective”[#1].*

*“…some (of the public) don’t even understand what you are talking about because you are saying priority setting. What is priority setting? Unless you say what are the issues that affect you in your community that you want us to research? Otherwise it’s a buzzword...” [#23].*

Subsequently, respondents discussed the need for; and a lack of tools through which the public interests can be identified and considered in the prioritization process, so as to increase the public’s acceptability of the priority setting decisions;

*“…There’s always this tug of war with the people. You feel they should be involved; (however), we need a tool that collects what affects (the community) on a daily basis. We should develop a mechanism for collecting information and setting priorities that will be accepted by the public…” [#3].*

Furthermore, while some of the respondents thought that health research prioritization in Zambia was participatory, involving a variety of stakeholders across, age, gender and area of residence, some of the other respondents felt that certain groups such as the community, young professionals, people from rural areas, and women were still poorly represented;

*“…I felt good about the meetings and people attending but the part that troubles me is that it was nearly like parliament. There are front benchers and back benchers. You know, there’s a second row where the younger, inexperienced people sit and the older people, the professors, the heads of organizations take the front bench. So maybe it’s not as participatory as I feel but that’s sort of how it’s done...*” [#7].

*“Basically I think there were more men than women.”* [#3].

## Discussion

The paper presents findings from a study on health research priority setting and specifically, stakeholder involvement in health research priority setting in Zambia. While the technical stakeholders, policy makers and funders are reportedly involved in the process, the ultimate beneficiaries---the community/ public—was reportedly not well represented in the process. There was also poor representation along the age, area of residence and gender dimensions.

The findings that health research funders seem to play a big role in determining health research priorities is not surprising and are consistent with the literature on stakeholder engagement in decision making. Studies on priority setting in similar settings have identified this as a critical issue. While it is recognised and appreciated that the funding agencies are accountable to their parent organizations, the legitimacy of their influencing the priorities in LICs have been questioned [16]. Specific to health research, some studies have discussed how it is seems futile to set priorities at the national level, since countries are unable to fund the priorities and the agenda of what research is conducted ends up being opportunistically determined—and in accordance to the funding agencies' priorities [4, 17, 18]. This is not problematic if research funders’ priorities are consistent with the national priorities; problems arise when there are inconsistencies. Funding priorities outside those identified by the national research organizations inevitably affects the kind of evidence that is generated and is available to support evidence based decision making.

Respondents recognized government- specifically the ministry of health- as the legitimate lead stakeholder in health research prioritization. Their legitimacy arises from being the main users of the generated evidence; as well as their technical understanding of the research issues. Researchers were also identified as legitimate stakeholders since they have the skills for conducting health research. These findings are consistent with the literature [19]. However, while respondents identified the community/public/ patients as having the legitimacy to participate in health research priority setting since they are “the ultimate” beneficiaries of the research. The reasons that the respondents gave for the relevance of community/ public/ patient involvement are consistent with the literature [4, 12] and should ideally, motivate the facilitators of the prioritization process to involve this group. However, this does not seem to be happening. Although unfortunate, this finding is not surprising and has been widely documented both in priority setting for health research and health interventions [2, 4, 10, 12].

Some of the barriers to community participation identified by the respondents; including the community’s lack of understanding of priority setting which was perceived as technical (which may hinder their meaningful engagement in the deliberations), limited time and financial resources, limited skills in how to meaningfully engage with this group of stakeholders, have been described in similar contexts and need to be addressed in order to facilitate meaningful community engagement [20]. While cultural norms, (which marginalise certain groups such as young people and women); have been identified as barriers to participation in similar contexts [20], it was surprising that it was not identified as a barrier in this study. This could, in part, be explained by reporting bias or lack of awareness of this as a barrier by the respondents. Another issue arising from the results is the challenge of balancing between direct and representation participation and how to integrate community priorities and even at what point to engage the community [21]. Some have argued that it is impossible to directly involve communities, representatives such as civil society organizations, patient advocates are legitimized in some of the literature [22]. However, the degree to which politicians can represent community needs, has been questioned [20].

While the resources are limited; the benefits of involving the community/ public/patients in health research prioritization, as identified by the respondents, makes it imperative that Zambia endeavours to involve this group of stakeholders. Decentralized, bottom up health research priority setting whereby priorities are set at the community level, then fed into the sub- regional process, which in turn feeds into the national level process, could facilitate meaningful involvement [15, 20]. However, such a process would require skilled personnel, time and finances. Furthermore, the facilitators of the PS process should ensure that the community/public/patients participation is not tokenism. There should be explicit mechanisms through which the community priorities are actually integrated into the national level process. Mechanisms such as those recommended in the CHNRI and JLA approaches could provide guidance [1, 7, 8].

### Study limitations

*First*, this paper uses the terms community, public and patients interchangeably. This may be perceived as a limitation if one thinks about health research priority setting for a particular disease condition. However, the focus of this study was at the national level, which would include all possible researchable health issues. This means that all disease conditions could potentially be considered. Hence, it would be impossible to identify which specific patient group to include. In such a context, discussing the involvement of community/ public members seemed more appropriate since these represent all the potential patients.

*Second,* our sampling strategy did not lead us to any stakeholder from the community/public/patient. This meant that their lived experiences were not enlisted in the paper. However, this may be a reflection of the reality discussed by the respondents that the community is often not involved in the prioritization process. Future research should seek the views of the public.

## Conclusions

This paper presented findings from a study on stakeholder involvement in health research priority setting. While there is an intention to meaningfully involve various stakeholders in the prioritization process, this is not always realized. The technical stakeholders from the ministry of health and research institutions were thought to be legitimate leaders of the process, although there was a feeling that the research funders disproportionately influenced both the process and the kind of research that gets to be funded. There was limited participation of the public.

To facilitate participatory health research priority setting in Zambia, the health research funders need to commit to support the national health research agenda, this could be facilitated by their direct involvement in the prioritization process, followed with an incentive mechanism to ensure funding compliance. Since most of the research funding in LICs is investigator driven and comes from outside sources, countries should collaborate to ensure that even the external calls highlight the locally identified research priorities.

Facilitators of health research prioritization need to explore feasible mechanisms for meaningful public engagement. Decentralized priority setting could be one avenue [15]. Other mechanisms could include enlisting and considering public values in the process, or publishing the identified priorities and facilitating public dialogue on the identified priorities and responding to any contrary priorities that may arise as a result of this dialogue.

## Abbreviations

CAM: The Combined Approach Matrix
CHNRI: Child Health and Nutrition Research Initiative
CSO: Civil society organizations
ENHRS: The Essential National Health Research Strategy
JLA: The James Lind Alliance
L4D: Listening for Direction
LRG: Larger Reference Group
NGO: Non-government organizations
PS: Priority setting
TWG: Technical Working Group
